# Enhancing recruitment methodologies: leveraging the Tailored Design Method to survey populations with varied engagement in healthcare

**DOI:** 10.1186/s12874-026-02895-0

**Published:** 2026-06-09

**Authors:** Hari H. Venkatachalam, Nina A. Sayer, Heather Belanger, Peter A. Toyinbo, Stephen L. Luther, Shannon R. Miles

**Affiliations:** 1 Research and Development Service, James A. Haley Veterans’ Hospital, Tampa, FL USA; 2 Tampa VA Research and Education Foundation, Inc., Tampa, FL USA; 3 Department of Epidemiology, Gillings School of Global Public Health, University of North Carolina at Chapel Hill, Chapel Hill, NC USA; 4 Center for Care Delivery and Outcomes Research, Minneapolis VA Health Care System, Minneapolis, Minnesota USA; 5 Departments of Medicine and Psychiatry, University of Minnesota, Minneapolis, USA; 6 Department of Psychology, University of South Florida, Tampa, Florida USA; 7 Department of Psychiatry & Behavioral Neurosciences, Morsani College of Medicine, University of South Florida, Tampa, Florida USA; 8 College of Public Health, University of South Florida, Tampa, Florida USA; 9 Center of Innovation for Complex Chronic Healthcare, Hines, IL USA; 10 Mental Health and Behavioral Sciences, James A. Haley Veterans’ Hospital, Tampa, Florida USA; 11 Department of Neurosurgery, Brain and Spine, University of South Florida, Tampa, Florida USA

**Keywords:** Survey methods, Internet survey, Postal survey, Survey methodologies in health research, Response rate

## Abstract

**Background:**

Successfully recruiting respondents to complete surveys is integral for ensuring representative samples and providing adequate power for analyses. However, obtaining survey responses for public health and health services research can be challenging, especially when working with populations with varied engagement with the providers of the institution conducting the study. Recruitment may be particularly challenging when selecting potential participants based on historical medical encounters that they may not remember, such as a brief health screen. Many researchers implement the Tailored Design Method (TDM) to optimize response rates. Adaptations to TDM, however, may be needed for researchers in federal institutions conducting surveys of populations with varied levels of healthcare engagement. This manuscript describes TDM implementation techniques and modifications that comply with federal regulations, and factors associated with response rates and survey response type.

**Methods:**

We surveyed a random, stratified sample of 9,775 U.S. Iraq and Afghanistan war veterans whom the Veterans Health Administration (VHA) had screened between 2007 and 2018 for post-deployment health concerns. We implemented a modified TDM that complied with federal regulations to survey veterans by email and USPS between July 2020 and August 2021.

**Results:**

Twenty-one percent (*N* = 2,025) responded. It took a median of 21 and 69 days to receive responses via electronic survey and paper booklets, respectively. The random sample of veterans had varied levels of engagement with the VHA at the time of health screening and survey. Respondents and non-respondents used equal amounts of neurology, physical medicine and rehabilitation, and mental health VHA services in the 6 months post-screens. At the time of the survey (an average of 9 years post-screen), 21% of respondents reported not having recently used VHA services. Respondents were more likely to be older, White, and female than non-respondents, although differences were small in magnitude. Compared to electronic survey respondents, paper booklet respondents were more likely to be older, rural, and screened earlier, and less likely to live in the same state as the research team. When controlling for first contact mode (email vs. USPS), only age predicted whether veterans responded by paper booklet or electronic survey.

**Conclusions:**

Investigators working in federal organizations should be aware of possible lower response rates when compared to surveys of civilians. Researchers in federal institutions seeking to optimize response rates may find our TDM adaptations useful when surveying historical cohorts or populations without regular interaction with the research institution. Our response rate (~ 21%) may be useful for researchers conducting sample size calculations with similar populations.

## Introduction

Two main challenges hinder successfully recruiting individuals to respond to research surveys. First, researchers need to recruit enough respondents to sufficiently power analyses. Second, researchers must ensure that respondents are a representative sample to limit biases in the research findings [1–4]. The Total Design Method, originally conceived by Don A. Dillman in the 1970s, created guidelines for survey collectors to obtain high response rates and representative samples [5].

Dillman’s techniques are rooted in social exchange theory and strive to improve survey response rates by removing costs and barriers while enhancing benefits and building trust [6]. The growth in electronic surveying options prompted modifications to the Total Design Method. The most recent iteration of the technique, termed the Tailored Design Method (TDM), allows for surveying through both postal service (e.g., USPS) mail and email channels [6]. Key characteristics of TDM include sending a token cash incentive with the survey request, personalizing communication with handwritten signatures, and providing individuals with both electronic and paper-based options to complete the survey [6, 7]. Other recommendations include tailoring succinct, direct, and user-friendly questionnaires that reduce respondent burden [6].

Strict implementation of Dillman’s recommendations has resulted in survey response rates greater than 70% in some civilian samples [8–12]. However, other studies have noted lower response rates between 12 and 40% in veteran samples and / or samples of individuals who did not have an ongoing relationship with the surveying institution [1, 13–18]. Lower response rates in some of the studies may occur due to a variety of factors, including regulatory constraints that prevent research institutions from implementing certain features of TDM. Within U.S. government institutions, sending cash incentives to all individuals has tax implications on income-based government benefits [19]. Instead, Institutional Review Board-approved research studies traditionally only provide incentive payments post hoc to those who complete a survey [20]. Sending post-survey incentive payments rather than initial token payments with the survey request has been shown to reduce response rates in surveyed samples, including veterans [7, 13]. There are also resource constraints on personalizing communication when performing large survey mailouts. Additionally, standardized self-report measures of health outcomes included in surveys may not be succinct or user friendly. These factors may have contributed to lower response rates for surveys administered by the US Veterans Health Administration (VHA) compared to surveys of civilians.

Another consideration for TDM implementation is how to reach a representative sample of participants who have ever used the healthcare system, and not just those currently engaged in healthcare. VHA research studies typically seek respondents who are engaged in VHA healthcare. This often includes restricting samples to individuals who had recent diagnoses or encounters with their healthcare provider [10, 14, 16]. However, only surveying individuals who recently engaged in VHA healthcare limits the generalizability of the findings to recent VHA users. VHA performs research in a variety of domains, such as satisfaction with care, determinants to seeking care, and quality of life studies that would benefit from the participation of populations with varied levels of engagement in healthcare [21–23]. Surveying both individuals who are engaged and those who are less engaged in receiving VHA services can provide important information to inform quality improvement projects, especially initiatives to increase service utilization among non-users [24].

This paper presents the survey methods of a large, national VHA study that sought to examine clinical outcomes of Afghanistan and Iraq war veterans who underwent the VHA-mandated post-deployment screenings for Traumatic Brain Injury (TBI) and Mental Health (MH) conditions between 2007 and 2018 [25–27]. We selected a random stratified sample of individuals based on a specific historical anchor timepoint: the date VHA screened the veterans for TBI and MH conditions. This anchor timepoint preceded the survey data collection by up to twelve years. Because current engagement in VHA was not an inclusion criterion, those surveyed may not have been invested in VHA clinical care or research. Within this manuscript, we describe how we implemented TDM with adjustments for VHA context and regulations. To determine if we selected a representative sample with these methods, we examined differences between survey responders and non-responders. Additionally, we assess demographic differences by survey response type (i.e., paper booklet vs. electronic survey). Our goal is to inform other survey studies that are conducted by large and / or federal healthcare organizations, and who seek to survey groups of individuals with varying levels of engagement with these organizations.

## Materials and methods

### Human ethics and consent to participate

The study was approved by the Department of Veterans Affairs and Institutional Review Board. The study adheres to all state and federal ethical research guidelines. All respondents completed altered informed consent either electronically or on paper in the survey booklet prior to completing any study related activities. A clinical trial number was not applicable for this research study.

### Sample selection

To meet the aims of the parent study, we selected veterans from a population of VHA patients who completed a TBI screen between 10/01/2007 and 9/30/2018 and a MH screen within 7 days of the TBI screen (*N* = 289,104) [26]. The VA implemented the TBI screen in 2007, and the parent study was funded in 2018. From this population, we selected a random sample of 9,775 veterans. To meet the objectives of the parent study [26], we generated the sample stratified by MH screen outcome (screened positive for: posttraumatic stress disorder, depression, and / or alcohol use disorder), TBI screen outcome (probable TBI vs. no TBI), biological sex (male or female), VHA region (i.e., North Atlantic, Southeast, Midwest, Continental, and Pacific), and fiscal year of TBI screen (i.e., grouped into three cohorts of FY 2008–09, FY 2010–12, and FY 2013–18).

### Data sources

To advance both research and medical care, VHA has developed large databases that house administrative data (e.g., diagnoses, types and frequency of medical visits, demographics) for all veterans in the national healthcare system. The current study paired administrative data with a survey that evaluated current functioning and satisfaction with VHA healthcare [26, 28]. We extracted from the administrative datasets TBI screen results; demographic variables of age, biological sex, race, and ethnicity; service utilization in the form of visit counts (i.e., Stop codes) in the domains of physical medicine and rehabilitation (PM&R), neurology, and mental health (MH); and email addresses, residential addresses, and telephone numbers for each veteran [28, 29]. Within the VHA administrative datasets, we drew demographic variables from the VA’s Observational Medical Outcomes Partnership datasets. We drew patient contact information from the Spatient domain. We drew TBI Screen results from the VA TBI Screening Registry datasets.

Demographic variables and contact information were extracted from the VHA databases at the time of the survey data collection. We assessed VHA service utilization at two time points. First, contemporaneous with TBI screening using the administrative dataset’s outpatient domain, and second, from respondents, at the time of the survey response via self-report [26, 28].

We validated contact information (i.e., email addresses, residential addresses, phone numbers) for contacted individuals by using the most recently updated active record in the VHA administrative datasets. We assessed rurality of home residence based on zip codes extracted from the residential addresses and evaluated against a crosswalk generated by the Washington, Wyoming, Alaska, Montana, Idaho’s Rural Health Research Center Rural-Urban Commuting Area Codes [30]. 

### Modified TDM contact schedule

**Fig. 1:**
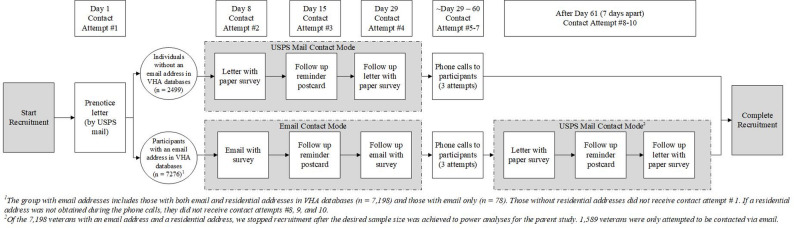
Survey recruitment methodology through a modified Dillman (TDM) method

The research team attempted to survey 9,775 individuals from the stratified random sample between July 2020 and August 2021. We ceased recruitment efforts after we reached the sample size necessary to power analyses for the parent study [26]. Recruitment consisted of up to ten contact attempts performed via multiple contact modes: emails sent through the Qualtrics platform (i.e., email mode), packets sent through USPS mail (i.e. USPS mail mode), and / or telephone calls (See Fig. 1). In line with TDM recommendations, we attempted to use all three contact modes to contact individuals. However, 26% of those selected for recruitment were missing either an email or a residential address in VHA databases. While most individuals in the stratified random sample had both email and residential addresses in the administrative datasets (*n* = 7,198), 78 had only email addresses, and 2,499 had only residential addresses. We attempted to obtain missing email addresses and residential addresses through phone calls with non-responders. Ultimately, we attempted to contact 3,304 veterans by email only, 2,494 by USPS mail only, and 3,977 by both email and USPS mail. While we did not attempt to contact 1,589 email mode non-respondents with residential addresses by USPS mail, we estimated the number of additional responses using response rates from those attempted to be contacted using both modes.

If the research team had both an email and residential address, we completed the email contact attempts prior to initiating USPS mail contact attempts for non-responders. Contact attempts consisted of: (Contact attempt #1) a pre-notice letter if veterans had a residential address; (Contact attempt #2) an email or USPS mailed letter with the survey; (Contact attempt #3) an electronic or USPS mailed opt-out postcard; and (Contact attempt #4) a follow-up email or USPS mailed letter with the survey packet. After completing all attempts with the first contact mode (email or USPS mail), we attempted three telephone calls (Contact attempts #5–7) to confirm receipt of the survey and to address any questions or concerns. Subsequently, the letter and survey, opt-out postcard, and follow-up letter and survey were repeated via the USPS mail mode for non-respondents who had both email and residential address contact information (i.e., Contact attempts #8–10). A small subset (*n* = 78) had only email addresses and therefore did not receive the pre-notice letter. If a residential address was not obtained during phone calls, non-responders from this subset were not contacted at any point by the USPS mail mode.

Survey respondents could complete the survey in either a paper booklet or an electronic survey. We provided multiple survey response options at each contact attempt. For example, in the letter attached to the paper booklet version of the survey sent via USPS mail, there was a shortened URL and a QR code to access the electronic survey. Emailed veterans received email-embedded links to the electronic survey and the opportunity to request a paper booklet. All paper surveys included self-addressed pre-paid return envelopes for the respondents’ convenience. Contact attempts ceased after the individual completed the survey, opted out, was identified as deceased, or when the email and / or USPS mail contact attempts were completed.

### Modifications to TDM

Consistent with TDM, we generated visually appealing contact letters and paper booklets with the assistance of a professional graphics team. Department of Veterans Affairs logos and branding were used to improve the visual appeal of the booklet and build trust. We incorporated feedback from a test group of veterans on elements such as the readability of the survey (e.g., font size, contrast, ease of following directions) and preference for the cover page design.

Key modifications of TDM for VHA regulatory requirements include retaining survey instruments in their validated forms and sending gift cards only to survey respondents, rather than sending incentives to all individuals we attempted to contact. A complete list of TDM implemented features and adaptations is listed in Table 1 [6, 31].

**Table 1:** Implemented features of the modified TDM

TDM implemented features and adaptations for VHA Survey
**Survey layout strategies**
Created a user-friendly booklet for the survey, with a double-stapled binding.
Used validated instruments in surveys and retained matrix-style question layout formats. Asked each instrument sequentially.
Instructions for the survey were short and located on the inside cover of the booklet (i.e. ,page 2) and on the first screen of the electronic survey.
The Informed Consent document was separate from the survey. Individuals could access it through a link or on pages separate from the survey. Instructions for individual measures were included on the same page as the question items.
Used 14 + font sizes and bolded the questions. Used instrument names to separate groups of questions. Increased font contrast on the electronic survey to improve the readability on phones. Institutional graphic artists developed several customized covers for the survey, which were tested with a group of veteran stakeholders.
All electronic surveys had “back buttons” so respondents could return to previously answered questions.
Respondents were able to leave questions blank on both the paper and the electronic surveys, but we did not include a “Refuse to answer” option.
Added conditional logic to electronic surveys to skip irrelevant questions. On the paper survey, the survey text prompted respondents to skip questions that were not relevant to them.
The final page/screen only contained a thank-you message and two organizational logos.
**Quality control and administrative strategies**
Performed literature reviews for best practices for TDM implementation.
Undeliverable USPS mail or emails triggered stopping the respective contact modes. Phone calls were used to attempt to obtain current contact information. Team obtained preferred USPS mailing addresses for incentives through a question on the electronic survey. Team completed three follow up calls for any returned incentives.
Generated unique ID numbers to limit communication of any Protected Health Information and Personally Identifiable Information.
Created a Microsoft Access database for tracking progress through the recruitment protocol, opt-outs, respondents’ survey completion, and gift card delivery.
**Mail and email communication strategies**
Research team contacted individuals using USPS mail, email, and telephone. Email preceded USPS mail contact attempts.
Mailouts and emails occurred in spaced gaps of 1–2 weeks.
Individuals could respond to the paper surveys via self-addressed envelopes. Individuals could respond to electronic surveys via QR codes, shortened URLs, and links embedded in emails.
**Telephonic communication strategies**
Research team made three phone attempts, on varying days and times. Discontinued attempts if individual declined participation, hung up, or number was inaccurate. Calls staggered by 2–3 days and batched by individual’s time zone.
Utilized a detailed voicemail script. Generated protocols to handle suicide ideation. Recorded a personalized voicemail message for the study phone number.
**Trust building strategies / reducing response burden and costs**
Survey respondents received gift cards upon completing the survey, rather than using a token cash incentive.
Highlighted the importance of the research in contact letters, e.g. “This study will help the VA better understand how to improve healthcare for all veterans across the country.”
Letters contained personalized headers, e.g. “Dear John Doe,” accomplished through mail merge options in Microsoft Word. All letters thanked respondents for their time and assistance. Used an image of the Principal Investigator’s signature in blue ink on all communication.
Electronic surveying option provided to remove mailing burden. Paper booklet respondents were able to return surveys using pre-paid, self-addressed envelopes.
Survey could not be completely anonymized due to study needs to connect respondents to their health record. Ensured confidentiality of responses in all communication.
A test group (i.e. local Veteran Engagement Counsel) reviewed and provided feedback on the survey. Limited the first wave to 50 contacted individuals to limit the impact of any undetected errors.
Published results from the study and made them publicly available.

### Software systems

We used Qualtrics, a surveying platform that allowed customization (e.g., contact list generation) and automation (e.g., timed survey delivery), to email the electronic survey [32]. Streamworks, a vendor specializing in data digitalization, converted our paper booklets into a digital format for data collation [33]. We mailed the completed paper booklets to Streamworks for scanning and data storage in an electronic database.

### Analyses

Assessed characteristics were race (white vs. non-white/mixed race), biological sex (female vs. male), ethnicity (Hispanic vs. non-Hispanic), rurality (rural vs. urban), proximity to research site (Florida vs. non-Florida), age (at the time of survey data collection), and the number of years between the TBI Screen and the contact attempt. As missingness on covariates was less than 5%, we performed a complete case analysis on the dataset. To compare respondents to non-respondents, we performed two-sample t-tests for continuous variables and Chi-squared analyses for dichotomous variables. Additionally, we employed multivariable logistic regression models comparing respondents to non-respondents. Furthermore, we compared those who responded by paper booklet to those who responded by electronic survey. We performed two-sample t-tests and Chi-squared analyses among all respondents based on survey response type. Additionally, we employed multivariable logistic regression models comparing paper booklet vs. electronic survey respondents on demographics.

Adjusted odds-ratios for all predictor variables estimated the effect sizes. We assessed the magnitude of the effect sizes using cut points of 1.68, 3.47, and 6.71 for adjusted odds ratios to refer to small, medium, and large effect sizes, respectively [34]. Because the mode the survey was first sent to the veteran (email and then USPS mail vs. USPS mail only) may impact whether a person responded or how they responded (electronic survey or paper booklet), we adjusted these models with first contact mode and assessed changes to effect sizes after adjustment.

We conducted a number of exploratory analyses to determine if health factors and service utilization were related to response rates. To assess if contacted individuals who screened positive on the TBI screen would be more likely to respond, we performed Chi-squared analyses comparing respondents to non-respondents with TBI screen outcome (positive vs. negative). We additionally performed non-parametric statistical tests (i.e., Mann-Whitney U test) to determine if respondents and non-respondents had different service utilization around the anchoring timepoint. Also, to determine if our respondents included low utilizers of VHA care, we obtained descriptive statistics of VHA service utilization from survey respondents around the time of survey completion.

Finally, to assess the speed of response and the utility of the multiple touchpoints of our modified TDM, we performed descriptive statistical analyses on the time between survey sent and receipt of returned survey and the number of responses per day following each of the steps of the email contact mode.

## Results

**Fig. 2:**
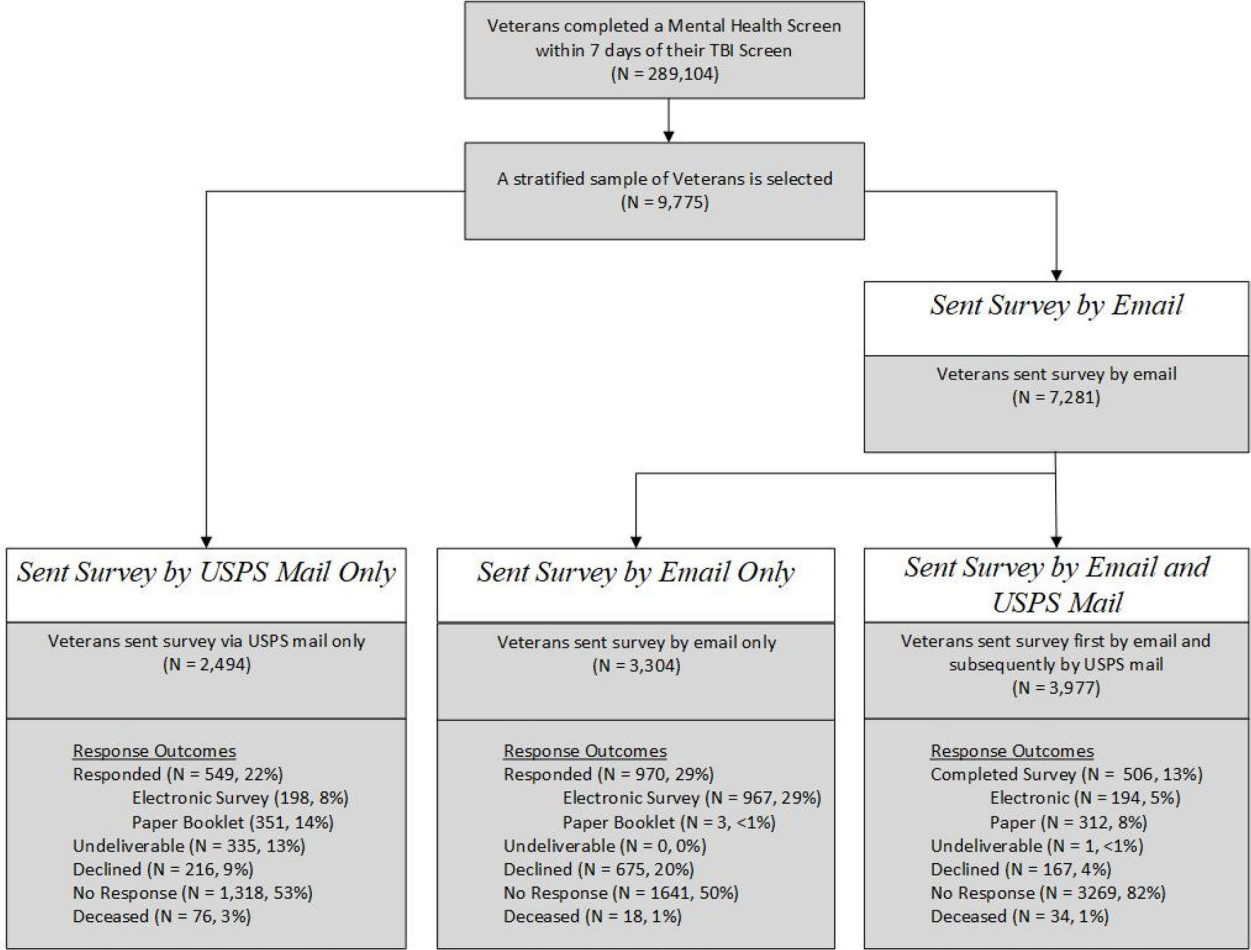
Flowchart and outcomes from contact mode

By the end of data collection, 2,025 (21%) of the stratified random sample of 9,775 veterans responded to the survey, and 1,058 (11%) actively declined participation in the study. Response rates differed across contact mode: 20% of veterans who were initially contacted by email mode and 22% of veterans initially contacted by the USPS mail mode completed the survey. Among those with both residential and email addresses, the majority of non-respondents (*n* = 3244, 67%) were contacted by both email and USPS mail modes (See Fig. 2). Most of the veterans we attempted to contact (*n* = 6,228, 64%) neither responded to the survey nor actively opted out of participating. Although we did not complete the USPS mail contact mode for 1,589 veterans with both an email and residential address, we estimate from response rates of participants contacted by both modes we would have obtained an additional 88 survey responses (i.e., < 1% change in response rate).

The research team made active efforts to remove deceased individuals from the recruitment pool through administrative data searches before contacts. Still, 128 (~ 1%) veterans died during the time between pulling their contact information from the administrative data and when we began the surveys. The team was informed of the veterans passing through email, mail, or phone call responses from their loved ones. Both USPS and email contact modes failed for an additional 336 veterans (3%). Invalid email and residential addresses were determined through bounced recruitment emails and returned USPS mail, respectively.

**Fig. 3:**
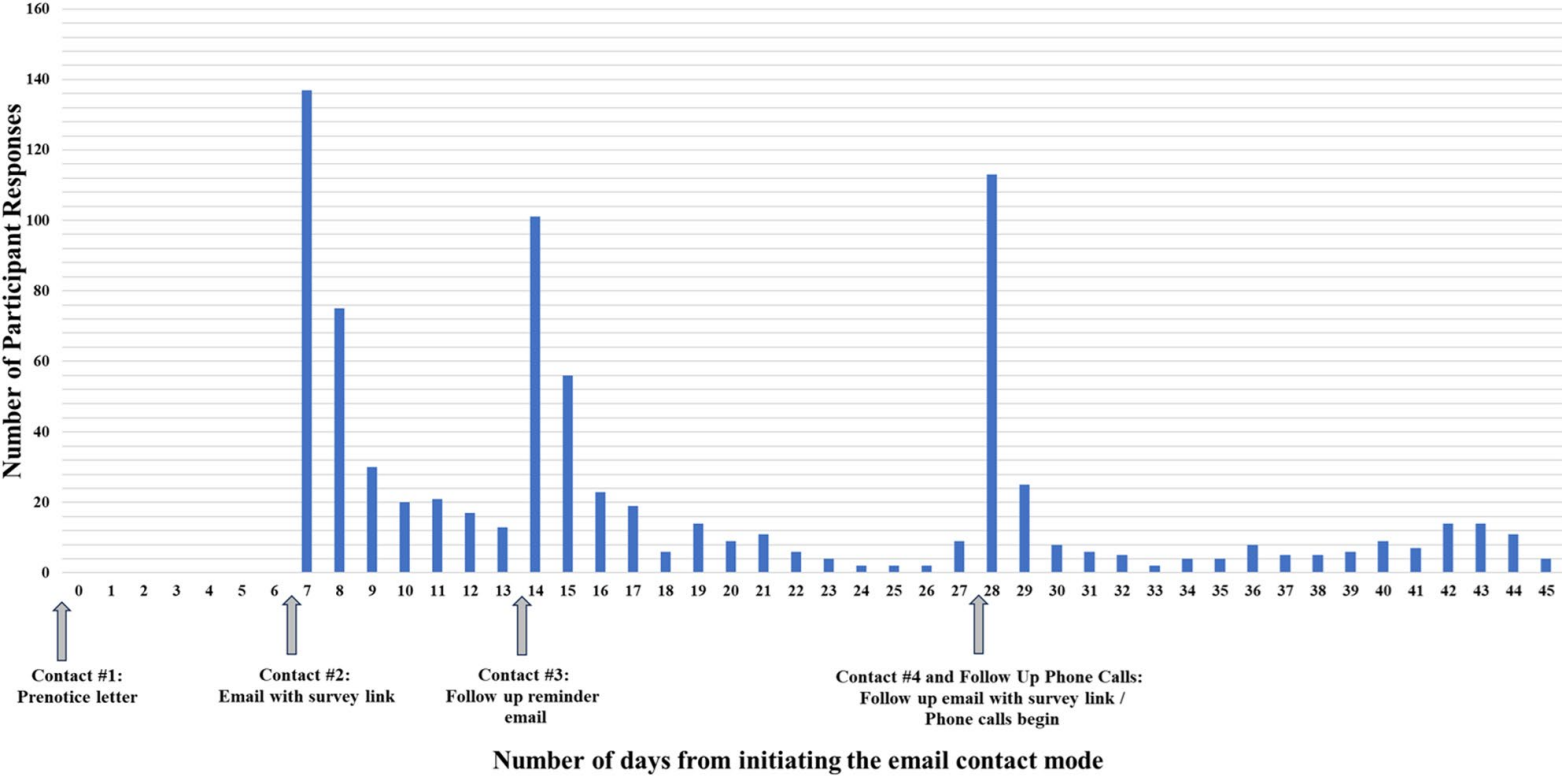
Electronic responses per day from the start of the email mode

We noted response rate spikes immediately after each email delivery of the email contact mode. Responses tapered slowly after each touchpoint (See Fig. 3). Spikes in responses were not as apparent after the USPS mail mode contact points.

As expected from having sent surveys by email first, more respondents (67%) responded via the electronic survey than the paper booklet. Also, unsurprisingly, the research team received electronic survey responses quicker than paper booklet responses. The median time for an electronic survey response was 21 days (IQR: 5–57 days) from first sending the survey (i.e., Contact #2), while the median time to receive paper booklets was 69 days (IQR: 42–123 days) from first sending the survey.

Bivariate analyses showed respondents were more likely to be White (χ^2^ = 11.38, *p* < 0.01), female (χ^2^ = 14.13, *p* < 0.01), older (*t*-statistic = -12.12, *p* < 0.01), and screened earlier for post-deployment health concerns (*t*-statistic = -3.31, *p* < 0.01) compared to non-respondents (See Table 2). Bivariate analyses between survey response type showed those who responded by paper were more likely to be rural (χ^2^ = 7.83, *p* = 0.01), older (*t*-statistic = -7.88, *p* < 0.01), residents of a different state than the recruitment site (i.e., Florida) (χ^2^ = 9.29, *p* < 0.01), and screened earlier for TBI and MH conditions (*t*-statistic = -4.90, *p* < 0.01) compared to those who responded by electronic survey (See Table 3). The Chi-squared analysis comparing TBI screen outcome (positive or negative for TBI) with whether individuals responded was not statistically significant (χ^2^ = 1.42, *p* = 0.234).

**Table 2:** Demographic comparisons between survey responders and non-responders

			Survey Respondent vs. Non-respondent^1^		
Variable		Stratified Random Sample of Veterans **(*n*** **= 9734)**^**1**^	Respondent **(*n*** **= 7709)**	Non-respondent **(*n*** **= 2025)**	Statistical Test
Race	White	6619 (68%)	1440 (71%)	5179 (67%)	Χ^2^ (1, *N* = 9734) = 11.38, *p* = 0.001
	Non-White	3115 (32%)	585 (29%)	2530 (33%)	
Sex	Female	1422 (15%)	349 (17%)	1073 (14%)	Χ^2^ (1, *N* = 9734) = 14.13, *p* < 0.001
	Male	8312 (85%)	1676 (83%)	6636 (86%)	
Ethnicity	Hispanic	1160 (12%)	225 (11%)	935 (13%)	Χ^2^ (1, *N* = 9358) = 1.87, *p* = 0.172
	Non-Hispanic	8198 (88%)	1733 (89%)	6465 (87%)	
Rurality	Rural	1831 (20%)	405 (21%)	1426 (19%)	Χ^2^ (1, 9379) = 1.81, *p* = 0.179
	Urban	7548 (80%)	1562 (79%)	5986 (81%)	
Age	Age at Survey	42.82 ± 10.83	45.52 ± 11.44	42.11 ± 10.55	*t*(2989.9)^1^ = -12.12, *p* < 0.001
Proximity to Mailout Site	Florida	843 (9%)	184 (9%)	659 (9%)	Χ^2^ (1, 9633) = 0.39, *p* = 0.532
	Non-Florida	8790 (91%)	1838 (91%)	6952 (91%)	
Time between Screen and Survey	Years	9.08 ± 2.97	9.27 ± 2.94	9.03 ± 2.98	*t*(9728) = -3.31, *p* = 0.001

^1^Individuals deceased by first contact attempt removed from table

**Table 3:** Demographic comparisons between paper vs electronic survey responders

Variable		Paper Booklet (*n* = 666)	Electronic Survey (*n* = 1359)	Statistical Test
Race	White	464 (70%)	976 (72%)	Χ^2^ (1, *N* = 2025) = 1.00, *p* = 0.316
	Non-White	202 (30%)	383 (28%)	
Sex	Female	101 (15%)	248 (18%)	Χ^2^ (1, *N* = 2025) = 2.98, *p* = 0.084
	Male	565 (85%)	1111 (82%)	
Ethnicity	Hispanic	69 (11%)	156 (12%)	Χ^2^ (1, *N* = 1958) = 0.43, *p* = 0.514
	Non-Hispanic	569 (89%)	1164 (88%)	
Rurality	Rural	157 (24.23%)	248 (19%)	Χ^2^ (1, *N* = 1967) = 7.83, *p* = 0.005
	Urban	491 (76%)	1071 (81%)	
Age	Age at Survey	48.50 ± 12.49	44.06 ± 10.60	*t*(1147.3)^1^ = -7.88, *p* < 0.001
Proximity to Mailout Site	Florida	42 (6%)	142 (10%)	Χ^2^ (1, *N* = 2022) = 9.29, *p* = 0.002
	Non-Florida	623 (94%)	1215 (90%)	
Time between Screen and Survey	Years	9.71 ± 2.66	9.06 ± 3.04	*t*(1489.3)^1^ = -4.90, *p* < 0.001^2^

^1^Satterthwaite approximation of degrees of freedom for unequal variances utilized

Multivariable logistic regression models revealed similar results as the bivariate analyses with respondents being more likely to be female (aOR 1.40, 95% C.I. 1.22, 1.61), older (aOR per 5-year increment, 1.16, 95% C.I. 1.13, 1.19), and White (aOR for White respondents 1.31, 95% C.I. 1.16, 1.47) compared to non-respondents (See Table 4). After adjustment for first contact mode, predictors for respondents remained unchanged with sex (aOR for female sex 1.40, 95% C.I. 1.23, 1.62), age (aOR per 5-year increment, 1.15, 95% C.I. 1.13, 1.18), and race (aOR for white race 1.31, 95% C.I. .16, 1.47) remaining significant predictors of those who responded to the survey.

**Table 4:** Multivariate logistic regression models predicting survey response over non-response

Parameter	*DF*	Models (without adjusting for first contact mode)			Models (adjusting for first contact mode)		
		**β Estimate**	**O.R. and** **95% Wald C.I.**	***p*** **-value**	**β Estimate**	**O.R. and** **95% Wald C.I.**	***p*** **-value**
Intercept	1	-2.587		< 0.001	-2.520		< 0.001
Race (White vs. Non-White)	1	0.268	1.307 [1.163, 1.469]	< 0.001	0.267	1.305 [1.466, 1.163]	< 0.001
Sex (Female vs. Male)	1	0.339	1.404 [1.221, 1.613]	< 0.001	0.342	1.408 [1.225, 1.617]	< 0.001
Ethnicity (Hispanic vs. Non-Hispanic)	1	-0.129	0.879 [0.744, 1.039]	0.132	-0.121	0.886 [0.749, 1.047]	0.156
Rurality (Rural vs. Urban)	1	0.032	1.033 [0.907, 1.176]	0.624	0.023	1.024 [0.899, 1.165]	0.725
Age at Survey (in 5-year increments)	1	0.030	1.161 [1.133, 1.189]	< 0.001	0.028	1.152 [1.125, 1.18]	< 0.001
Proximity to Research Site (Florida vs. Non-Florida)	1	-0.016	0.984 [0.821, 1.179]	0.860	-0.017	0.983 [0.821, 1.178]	0.856
Years between Screen and Survey (in 1-year increments)	1	0.000	1.000 [0.982, 1.019]	0.970	0.001	1.001 [0.983, 1.02]	0.890
First contact mode (Email vs. USPS mail)	1				-0.026	0.975 [0.866, 1.097]	0.670

Multivariable logistic regression models comparing paper booklet respondents to electronic survey respondents revealed that respondents from Florida (i.e., research site) were less likely to respond by paper (aOR 0.610, 95% C.I. 0.417, 0.894). Participants were more likely to respond by paper booklet if they were older (aOR per 5-year increment, 1.193, 95% C.I. 1.139, 1.249) and were residing in a rural zip code compared with an urban zip code (aOR 1.329, 95% C.I. 1.047, 1.688). However, after adding first contact mode into the regression models (email vs. USPS mail), only older age remained a significant predictor of who responded by paper booklet (aOR per 5-year increment, 1.174, 95% C.I. 1.117, 1.234). Veterans who were first contacted by email were less likely to respond by paper booklet (aOR 0.167, 95% C.I. 0.133, 0.209) (See Table 5).

**Table 5:** Multivariate logistic regression models predicting response by paper booklet versus electronic survey

Parameter	*DF*	Models (without adjusting for first contact mode)			Models (adjusting for first contact mode)			
		**β Estimate**	**O.R. and** **95% Wald C.I.**	***p*** **- value**	**β Estimate**	**O.R. and** **95% Wald C.I.**		***p*** **-value**
Intercept	1	-2.738		< 0.001	-1.096		< 0.001	
Race (White vs. Non-White)	1	0.002	1.002 [0.802, 1.253	0.985	0.085	1.088 [1.389, 0.854]	0.494	
Sex (Female vs. Male)	1	-0.113	0.893 [0.682, 1.168]	0.409	-0.082	0.921 [0.690, 1.230]	0.579	
Ethnicity (Hispanic vs. Non-Hispanic)	1	-0.033	0.968 [0.692, 1.355]	0.849	0.037	1.037 [0.723, 1.489]	0.842	
Rurality (Rural vs. Urban)	1	0.285	1.329 [1.047, 1.688]	0.020	0.186	1.204 [0.930, 1.560]	0.159	
Age at Survey (in 5-year increments)	1	0.035	1.193 [1.139, 1.249]	< 0.001	0.032	1.174 [1.117, 1.234]	< 0.001	
Proximity to Research Site (Florida vs. Non-Florida)	1	-0.494	0.610 [0.417, 0.894]	0.011	-0.349	0.706 [0.471, 1.057]	0.091	
Years Before Mailout (in 1-year increments)	1	0.042	1.043 [1.006, 1.081]	0.021	0.017	1.017 [0.979, 1.057]	0.380	
First contact mode (Email vs. USPS mail)	1				-1.792	0.167 [0.133, 0.209]	< 0.001	

In terms of service utilization after the TBI screen, non-respondents and respondents used similar rates of mental health, neurology, and PM&R services. For example, 75% of survey respondents had ≤ 5 MH visits after they were screened for TBI, while 75% of non-respondents had ≤ 4 MH visits in the six months after the TBI screen. Most (75%) survey respondents and non-respondents had < 1 PM&R visit and 0 neurology visits in the 6 months after the TBI screen.

Screening results on the TBI and MH screens also did not appear to influence if veterans responded to the survey. Differences in service utilization between survey respondents and non-respondents were also not significant when stratified by TBI or MH screen outcome, with alpha adjusted to 0.008 using Bonferroni correction (Results not shown). Among those who had a positive MH screen, most survey respondents and non-respondents had less than 9 MH visits in the six months post-screen. Among those who had a positive TBI screen, most survey respondents had less than 7 PM&R visits and 0 neurology visits in the six months post-screen.

In terms of service utilization 6 months prior to the survey, 51%, 77%, and 53% of respondents reported not receiving MH, neurology, and PM&R services, respectively. Additionally, 21% of survey respondents reported that they had not received any VHA healthcare services in the 6 months prior to completing the survey (See Table 6).

**Table 6:** Individuals’ engagement with VHA services

Variable	Non-respondents (*n* = 6,528)		Respondents (*n* = 1,741)		
	Mean ± SD	IQR	Mean ± SD	IQR	*p*
Engagement with VHA after TBI Screen (*n* = 8,269)					
Engagement with VHA Healthcare after TBI Screen					
MH visits 6 months after the screen	4.64 ± 15.24	0–4	4.26 ± 12.81	0–5	0.495^*1*^
Neurology visits 6 months after the screen	0.09 ± 0.49	0–0	0.09 ± 0.47	0–0	0.590^*1*^
PM&R visits 6 months after the screen	1.73 ± 5.32	0–1	1.70 ± 4.12	0–1	0.316^*1*^

Engagement with VHA prior to Survey Completion (*n* = 1,741)^1^					
Variable				N	% (excluding missing)
Did not receive any VHA services 6 months prior to survey				359	21%
Did not receive PM&R services 6 months prior to survey				918	53%
Did not receive MH services 6 months prior to survey				881	51%
Did not receive Neurology services 6 months prior to survey				1320	77%

^1^Mann-Whitney U Test

## Discussion

Collecting representative data and ensuring adequate sample sizes are important for building robust datasets and successfully completing research studies. Researchers widely cite TDM as an effective method for obtaining these goals, yet federal and regulatory requirements may limit the full implementation of TDM. Where possible, we implemented TDM in accordance with Dillman’s guidelines. We made modifications to TDM in certain circumstances to address human resource limitations and to ensure alignment with VHA regulatory requirements. The objective of this analysis was to examine response rates associated with implementation of TDM within a large, federal healthcare system, surveying respondents with varying levels of healthcare engagement selected from a historical anchor timepoint (i.e., mandatory TBI and MH screens).

TDM is based on social exchange theory that leverages trust and transactions. We noted that over one quarter of the sample did not have an email or residential address, reflecting lack of regular use of VHA. Likely because our sample was drawn from a population with varied engagement, and due to regulatory limitations that prevented strict implementation of TDM, our modified TDM resulted in a lower than expected response rate (21%) compared to what has been reported in studies of civilian patients engaged in healthcare [8–12]. Although we ceased recruitment upon reaching a sufficient sample size for the analyses of the parent study, we do not believe this would have resulted in a substantial deviation from the 21% response rate (i.e., < 1% change in response rate).

Even with the lower response rate compared to other surveys using TDM, we obtained a sample of the surveyed population that was representative in terms of demographics and VHA care utilization. Analyses showed the surveyed population had minimal engagement with MH, neurology, and PM&R within the VHA. There were no statistical differences in service utilization at the time of the TBI screen between survey respondents and non-respondents. In regards to the MH engagement, which exceeded neurology and PM&R engagement, the number of sessions would be insufficient for a full course of evidence-based psychotherapy for the disorders for which the veterans were screened (i.e., PTSD, depression, and alcohol use disorder) [35–37]. When examining service utilization at the time of survey completion, 21% of the respondents reported not having received any VHA services in the 6 months prior to completing the survey. These findings support that the surveyed sample had varied engagement with VHA care, and that respondents are representative of the surveyed population on service utilization characteristics. As the parent study sought to examine the long-term effects of the VHA TBI screening program [26], it needed feedback from both veterans engaged and not engaged in VHA care. Successfully obtaining responses from those with little or no engagement with VHA met the needs of the parent study.

Possible reasons for the lack of engagement with VHA at the time of the survey may be due to the fact that most of the veterans completed the TBI and MH screens as part of their transition to civilian life and while relatively new to VHA healthcare. Combat veterans who served in the wars in Afghanistan and Iraq are eligible for free VHA healthcare benefits for 10 years after discharge from the Department of Defense [38]. After 10 years, if the veteran does not have a VHA service-connected disability, they may be required to pay for some or all of their healthcare depending on other factors, such as whether they have other insurance. Veterans without VHA disabilities may have transitioned their healthcare to civilian providers after their healthcare benefits expired.

Although there were no statistically significant differences between respondents and non-respondents in service utilization around the anchor timepoint (i.e., TBI screen), there were statistically significant but small differences in demographics between survey respondents and non-respondents. The small effect size differences in demographics support the survey sample’s representativeness to the overall VHA population that had post-deployment screens.

There were small effect size differences in rurality, age, proximity to research site, and time between screen and mailout between those who responded via paper booklet compared to those who responded via electronic survey. Florida residents were 39% (95% CI: 11% − 58%) less likely to respond via paper survey than non-Florida residents. Rural residents were 33% (95% CI: 5% − 69%) more likely to respond by paper than urban residents. Internet connectivity and familiarity with the research institution may be possible reasons for these differences. However, the impact of rurality, proximity to research site, and time between screen and mailout on paper booklet vs. electronic response was reduced when models were adjusted for first contact mode. The mode in which the first contact was attempted was a strong predictor of whether the survey response was a paper booklet or electronic survey. Although veterans were given options on how they would like to respond, 4% of participants contacted by email first (*n* = 315) responded by paper booklet, while 14% of those contacted by USPS mail first (*n* = 351) responded by paper booklet. Demographic characteristics may have impacted the availability of contact information for veterans and therefore may still be meaningful for survey response type preference.

The parent study could not have reached the required sample size if the team did not send out paper surveys. Even with the electronic survey option, a third of respondents responded via paper booklet. Researchers should allow respondents to complete surveys through both paper booklets and electronic surveys to ensure that the research team obtains a representative sample. Researchers may also use non-random sampling techniques, such as quota sampling, to ensure that underrepresented groups respond. Finally, subgroup analyses and multivariable modeling approaches can be used to check for sample biases and to account for differences in response rates in models.

Our modified TDM balanced federal regulatory constraints with the benefits of TDM to obtain a response rate that was sufficient for the needs of our larger study, although lower than anticipated based on research conducted in civilian samples by researchers who could employ all aspects of TDM. We offer six lessons learned for future researchers conducting survey studies within large healthcare organizations that survey similar populations.

### Lesson 1: Plan for a response rate lower than TDM advertises if you are studying historical cohorts, historical anchoring events, or involve individuals who are not engaged in healthcare at the research institution

This study recruited veterans who had undergone clinical screenings two to twelve years prior to asking them to complete a survey. After receiving our survey, some individuals reached out to the study team and stated they had never been screened for TBI or MH conditions, although the screening results appeared in the administrative datasets. The longer the time between the anchor point (i.e., TBI screen) and surveys, the less likely the individuals are to remember being screened, especially if the contacted individual screened negative for the condition (i.e., Recall Bias) [39]. Our analyses, however, did not reveal an association between TBI screen outcome (positive vs. negative) and survey response. If recall of the screen influenced the survey response, it did so for the entire sample, and not only for those who screened positive for TBI.

Additionally, many veterans screened for TBI or MH conditions did not seek VHA services after their screens [28]. Therefore, estimating a lower response rate may be useful for researchers who are attempting to recruit those who completed health screens or medical procedures years prior to data collection and who are no longer affiliated with the research institution.

### Lesson 2: Save research team members’ time by automating or outsourcing tasks, including prioritizing email

Leveraging available resources helped with limited research staff. The use of Qualtrics and Streamworks provided the team with resources to efficiently implement the modified TDM. Qualtrics capabilities included generation of mailout lists and automated delivery of electronic surveys, removing the burden of having to individually email surveys. Streamworks coded paper surveys by scanning them electronically, allowing for seamless merging of the electronic survey and paper booklet datasets.

Collating mail and stuffing envelopes are time-consuming tasks. To improve efficiency, we limited the number of instances that the team had to collate mail and stuff envelopes by implementing the email contact mode prior to the USPS contact mode. Subsequently, we excluded respondents and those who opted out from the USPS contact mode. We were able to utilize VHA mailroom staff to collate and seal the prenotice letter and send the opt-out cards without assistance from the research team. This resulted in the research team only having to collate and process mailings for the second and fourth mailouts from the USPS contact mode.

### Lesson 3: Implement TDM’s multimodal and multiple touch-point communication approach

A key feature of TDM is using both paper and electronic survey options and using multiple contact modes. Our team used USPS mail, email, and telephone contact modes to obtain responses from individuals. While performing multiple mailouts using both email and USPS mail contact modes is time and staff intensive, this methodology also ameliorates some of the logistical challenges the team faced. For example, because we were working with a historical cohort, the research team did not have updated contact information for everyone. Using multiple contact modes minimized the number of individuals with no valid contact information (i.e., *neither* a valid residential address nor email address; 3%; *n* = 336).

Restricting the survey response options to electronic surveys and limiting communication to email contacts may be appealing to research teams with limited human resources. However, our large number of paper booklet responses reveals that giving the option to respond via paper booklet was key to ensuring our response rate. Although we first attempted to contact all individuals by email and all USPS mail contact modes offered the option to access the electronic survey, a third of respondents (33%) still chose to respond via paper booklet. Ensuring a paper booklet option for survey response may allow representation from groups that may have connectivity issues with electronic surveys. Furthermore, the multiple touchpoints for the USPS mail and email contact modes were required to achieve our response rates. We noted a spike in response after each of the email touchpoints. Such bursts were harder to identify with the USPS contact mode, due to mail delivery lag. The findings from the email contact mode highlight the fact that repeated touchpoints resulted in increases in responses.

### Lesson 4: Follow Dillman’s recommendation for visual appeal of the survey

Dillman highlights visual appeal to encourage responses. Certain elements, such as an ink signature, were difficult to implement with the large number of USPS mailouts. Instead, we utilized an image of a blue color signature block from the Principal Investigator on USPS mailouts and the email signature line. We used an iterative process with input from multiple stakeholders to ensure a clear layout and visual appeal of the survey.

### Lesson 5: Establish an efficient process for incentive payments for respondents

Although our team was unable to provide an initial token incentive payment for all potential participants, we were able to establish an efficient process for paying respondents post-hoc. The survey requested respondents enter their preferred delivery address for their gift card, which ensured that they would receive payment. An internal tracking database assisted the team ensure all respondents received the incentive. We also had established procedures for returned incentives, including calling the respondent to ensure the team had the correct residential address.

### Lesson 6: Follow Dillman’s recommendations for building trust and increasing rewards

Our research team used Dillman’s overarching social exchange theoretical model to inform decisions we made throughout the study. Ensuring transparency of the research process and following regulatory requirements were central to building trust. We provided payments in a timely manner and reiterated at each touchpoint that we ensured anonymity of individual data in any results we published.

### Limitations and strengths

Following a modified TDM, we achieved a 21% response rate, which provided sufficient power for our analyses but is lower than previously published studies in other populations. While results demonstrated that certain characteristics had statistically significant associations with whether a veteran responded to the survey or not, or responded via paper booklet or electronic survey, the effect sizes were generally negligible. Our current method may be helpful for researchers conducting surveys in large and / or federal healthcare organizations but may be less relevant for researchers in other settings that have more flexibility to strictly follow TDM. Additionally, as our findings were related to veteran response rates, federal healthcare research in civilian populations may result in differing response rates.

Future research into response rates would benefit from experimental designs in which contacted individuals receive random assignment to one or both email and USPS mail contact modes. Concise covariate information (e.g., more specific racial categorizations) may provide clarity in demographic factors that impact response rates. Furthermore, future analyses would benefit from a priori generated causal models (e.g., DAGs) to determine confounders and mediators of the relationship between participant characteristics and response. Additionally, researchers would benefit from assessing service utilization at the time of survey completion for non-respondents, as that data was unavailable from the parent study.

### Future applications

Surveying is an important part of research activities. Gathering a sufficiently sized and representative sample is a central concern for any researcher who uses surveying for their study. Our goal was to assist researchers who are planning future studies using our protocol as a template for implementing TDM, especially when asking questions about historical anchoring events and surveying large populations of individuals who may not be engaged in healthcare services.

## Data Availability

Specific data from this study are not available.

## References

[1] Bayley PJ, Kong JY, Helmer DA, Schneiderman A, Roselli LA, Rosse SM, et al. Challenges to be overcome using population-based sampling methods to recruit veterans for a study of post-traumatic stress disorder and traumatic brain injury. BMC Med Res Methodol. 2014;14(48):1–9. 24713131 10.1186/1471-2288-14-48 PMC4101880

[2] Harrington KM, Nguyen XMT, Song RJ, Hannagan K, Quaden R, Gagnon DR, et al. Gender differences in demographic and health characteristics of the Million Veteran Program cohort. Womens Health Issues. 2019;25(S1):S56–66. 10.1016/j.whi.2019.04.012 PMC7061933 31253243

[3] Luoto TM, Tenovuo O, Kataja A, Brander A, Öhman J, Iverson GL. Who gets recruited in mild traumatic brain injury research? J Neurotrauma. 2012;30(1):11–6. 22909262 10.1089/neu.2012.2611

[4] Jang M, Vorderstrasse A. Socioeconomic status and racial or ethnic differences in participation: web-based survey. JMIR Res Protoc. 2019;8(4):e11865. 30969173 10.2196/11865 PMC6479282

[5] Hoddinott SN, Bass MJ. The dillman total design survey method. Can Fam Physician Med Fam Can. 1986;32:2366–8. PMC2328022 21267217

[6] Dillman DA, Smyth JD, Christian LM. Internet, phone, mail, and mixed-mode surveys: the tailored design method. 4th ed. Hoboken, New Jersey: Wiley; 2015.

[7] Millar MM, Dillman DA. Improving response to web and mixed-mode surveys. Public Opin Q. 2011;75(2):249–69.

[8] Al Khalaf K, O’Dowling Keane S, da Mata C, McGillycuddy CT, Chadwick BL, Lynch CD. Response rates to questionnaire-based studies in the contemporary dental literature: a systematic review. J Dent. 2022;126:104284. 36089221 10.1016/j.jdent.2022.104284

[9] Dillman DA, Dolsen DE, Machlis GE. Increasing response to personally-delivered mail-back questionnaires. J Off Stat. 1995;11(2):129.

[10] Kazzazi F, Haggie R, Forouhi P, Kazzazi N, Malata CM. Utilizing the Total Design Method in medicine: Maximizing response rates in long, non-incentivized, personal questionnaire postal surveys. Patient Relat Outcome Meas. 2018;9:169–72. 29922103 10.2147/PROM.S156109 PMC5995290

[11] Monroe MC, Adams DC. Increasing response rates to web-based surveys. J Ext. 2012;50(6). Available from: https://tigerprints.clemson.edu/joe/vol50/iss6/34/. Cited 2025 Nov 27.

[12] Smyth JD, Dillman DA, Christian LM, O’Neill AC. Using the internet to survey small towns and communities: Limitations and possibilities in the early 21st century. Am Behav Sci. 2010;53(9):1423–48.

[13] Coughlin SS, Aliaga P, Barth S, Eber S, Maillard J, Mahan CM et al. The effectiveness of a monetary incentive on response rates in a survey of recent U.S. veterans. Surv Pract. 2011;4(1). Available from: https://surveypractice.scholasticahq.com/article/3059-the-effectiveness-of-a-monetary-incentive-on-response-rates-in-a-survey-of-recent-u-s-veterans. Cited 2025 Nov 27.

[14] Culpepper WJ, Wallin MT, Magder LS, Perencevich E, Royal W, Bradham DD, et al. VHA multiple sclerosis surveillance registry and its similarities to other contemporary multiple sclerosis cohorts. J Rehabil Res Dev. 2015;52(3):263–72. 26220064 10.1682/JRRD.2014.07.0172

[15] Derrick JL, Eliseo-Arras RK, Hanny C, Britton M, Haddad S. Comparison of internet and mailing methods to recruit couples into research on unaided smoking cessation. Addict Behav. 2017;75:12–6. 28662435 10.1016/j.addbeh.2017.06.012 PMC5582000

[16] deRussy AJ, Jones AL, Austin EL, Gordon AJ, Gelberg L, Gabrielian SE, et al. Insights for conducting large-scale surveys with veterans who have experienced homelessness. J Soc Distress Homelessness. 2021;32(1):123–34. 10.1080/10530789.2021.2013013 PMC10208227 37234355

[17] Pflugeisen BM, Mou J. Patient satisfaction with virtual obstetric care. Matern Child Health J. 2017;21(7):1544–51. 28176034 10.1007/s10995-017-2284-1

[18] Vogt D, Perkins DF, Copeland LA, Finley EP, Jamieson CS, Booth B, et al. The veterans metrics initiative study of US veterans’ experiences during their transition from military service. BMJ Open. 2018;8(6):e020734. 29895650 10.1136/bmjopen-2017-020734 PMC6009506

[19] Largent EA, Eriksen W, Barg FK, Greysen SR, Halpern SD. Participants’ perspectives on payment for research participation: a qualitative study. Ethics Hum Res. 2022;44(6):14–22. 36316972 10.1002/eahr.500147 PMC9631331

[20] U.S. Department of Health and Human Services. Attachment A - addressing ethical concerns offers of payment to research participants. 2019. Available from: https://www.hhs.gov/ohrp/sachrp-committee/recommendations/attachment-a-september-30-2019/index.html. Cited 2025 Nov 28.

[21] Purcell N, Zamora K, Bertenthal D, Abadjian L, Tighe J, Seal KH. How VA Whole Health coaching can impact veterans’ health and quality of life: a mixed-methods pilot program evaluation. Glob Adv Health Med. 2021;10:2164956121998283. 33747639 10.1177/2164956121998283 PMC7940726

[22] U.S. Department of Veterans Affairs, Office of Research & Development. About the Office of Research & Development. 2025. Available from: https://www.research.va.gov/about/default.cfm. Cited 2025 Nov 28.

[23] Washington DL, Farmer MM, Mor SS, Canning M, Yano EM. Assessment of the healthcare needs and barriers to VA use experienced by women veterans: Findings from the National Survey of Women Veterans. Med Care. 2015;53:S23–31. 25767972 10.1097/MLR.0000000000000312

[24] Pietrzak RH, Southwick SM, Meffert BN, Morabito DM, Sawicki DA, Hausman C et al. US veterans who do and do not utilize Veterans Affairs health care services: Demographic, military, medical, and psychosocial characteristics. Prim Care Companion CNS Disord. 2019;21(1). Available from: https://www.psychiatrist.com/pcc/veterans-who-do-and-do-not-utilize-va-services. Cited 2025 Nov 27. 10.4088/PCC.18m02350 PMC6352911 30677266

[25] Returning home from Iraq and Afghanistan: Preliminary assessment of readjustment needs of veterans, service members, and their families. Washington, D.C.: National Academies Press. 2010. Available from: http://www.nap.edu/catalog/12812. Cited 2025 Nov 27. 25032369

[26] Miles SR, Toyinbo PA, Belanger HG, Venkatachalam HH, Luther SL, Sayer NA. Long-term clinical outcomes associated with the veterans health administration’s traumatic brain injury and mental health screens. J Head Trauma Rehabil. 2025;40(5):E349–59. 39969094 10.1097/HTR.0000000000001047

[27] U.S. Department of Veterans Affairs, Veterans Health Administration. Screening and evaluation of possible traumatic brain injury in operation enduring freedom (OEF) and Operation Iraqi Freedom (OIF) veterans. 2010. Available from: https://www.va.gov/optometry/docs/vha_directive_2010-012_screening_and_evaluation_of_possible_tbi_in_oef-oif_veterans.pdf. Cited 2025 Nov 28.

[28] Miles SR, Sayer NA, Belanger HG, Venkatachalam HH, Kozel FA, Toyinbo PA, et al. Comparing outcomes of the veterans health administration’s traumatic brain injury and mental health screening programs: types and frequency of specialty services used. J Neurotrauma. 2023;40(1–2):102–11. 35898115 10.1089/neu.2022.0176

[29] U.S. Department of Veterans Affairs. Traumatic brain injury screening and evaluation data. 2024. Available from: https://vaww.vhadataportal.med.va.gov/Data-Sources/Traumatic-Brain-Injury. Cited 2025 July 1.

[30] WWAMI Rural Health Research Center. 2005. Available from: https://depts.washington.edu/uwruca/ruca-data.php. Cited 2025 Nov 28.

[31] Dillman DA, Smyth JD, Christian LM. List of guidelines in internet, phone, mail, and mixed-mode surveys: the tailored design method, 4th edition. 2015. Available from: https://higheredbcs.wiley.com/legacy/college/dillman/1118456149/pdf/List_of_Guidelines_Internet_Phone_Surveys4e.pdf. Cited 2025 Nov 27.

[32] Qualtrics. 2025. Available from: https://www.qualtrics.com/. Cited 2025 Nov 27.

[33] Streamworks. LLC. Available from: https://streamworksmn.com/services/compliance-communications. Cited 2025 Nov 28.

[34] Chen H, Cohen P, Chen S. How big is a big odds ratio? Interpreting the magnitudes of odds ratios in epidemiological studies. Commun Stat - Simul Comput. 2010;39(4):860–4.

[35] U.S. Department of Veterans Affairs and U.S. Department of Defense. VA/DoD clinical practice guideline for the management of substance use disorders. 2021. Available from: https://www.healthquality.va.gov/guidelines/MH/sud/VADODSUDCPGProviderSummary.pdf. Cited 2025 Nov 28.

[36] U.S. Department of Veterans Affairs and U.S. Department of Defense. VA/DoD clinical practice guideline for the management of major depressive disorder. 2022. Available from: https://www.healthquality.va.gov/guidelines/MH/mdd/VADODMDDCPG_ProviderSummary_Final_508_updated.pdf. Cited 2025 Nov 28.

[37] U.S. Department of Veterans Affairs and U.S. Department of Defense. VA/DoD clinical practice guideline: management of posttraumatic stress disorder and acute stress disorder. 2023. Available from: https://www.healthquality.va.gov/HEALTHQUALITY/guidelines/MH/ptsd/VA-DOD-CPG-PTSD-Quick-Reference-Guide.pdf. Cited 2025 Nov 28.

[38] U.S. Department of Veterans Affairs. VA priority groups. 2024. Available from: https://www.va.gov/health-care/eligibility/priority-groups/. Cited 2025 Nov 28.

[39] Althubaiti A. Information bias in health research: definition, pitfalls, and adjustment methods. J Multidiscip Healthc. 2016;9:211–7. 27217764 10.2147/JMDH.S104807 PMC4862344

